# Treatment of bipolar clavicle injury with internal plating: a case series and literature review

**DOI:** 10.1186/s12891-023-06126-1

**Published:** 2023-01-05

**Authors:** Dongxu Feng, Yong Liu, Zijun Li, Jie Huang, Mei Fan, Xiaomin Kang, Jun Zhang

**Affiliations:** 1 Department of Orthopaedic Trauma, Hong Hui Hospital, Xi’an Jiaotong University School of Medicine, Xi’an, Shaanxi, China; 2 Center for Translational Medicine, the First Affiliated Hospital of Xi’an Jiaotong University, Xi’an, Shaanxi, China

**Keywords:** Bipolar clavicle injury, Open reduction, Plate, Surgery

## Abstract

**Background:**

Bipolar clavicle injury is a rare injury involving any combination of dislocation and/or fracture at both ends of the clavicle. Most reports of bipolar clavicle injury have been based on a single case, and treatment of this injury remains controversial. The present study was performed to evaluate the efficacy of surgical management with internal plating for bipolar clavicle injuries.

**Methods:**

We performed internal plating to treat seven consecutive bipolar clavicle injuries with different injury patterns from May 2013 to June 2021. A clavicle hook plate was used for five sternoclavicular joint injuries (including a revision surgery) and three acromioclavicular joint dislocations, a T plate was used for one sternoclavicular joint injury, and an anatomic plate was used for one distal clavicle fracture. At follow-up, radiographs were assessed for bone alignment, joint congruity, fracture union or malunion, and implant failure or migration. Clinical evaluation included determination of the Disability of the Arm, Shoulder, and Hand (DASH) score; Constant–Murley score; visual analog scale (VAS) score; and complications.

**Results:**

The patients were regularly followed up after the operation, and functional parameters were assessed over time. At a mean follow-up of 28.1 ± 22.0 months, each fracture had solid bone union, and each dislocation showed no sign of recurrent instability. The mean shoulder forward flexion was 159.3° ± 7.9°, and the mean DASH score was 8.8 ± 5.1. The mean Constant–Murley score was 88.9 ± 7.9, with six cases assessed as excellent and one case assessed as good. The mean VAS score was 1.0 ± 1.5, and the mean patient satisfaction score was 9.3 ± 0.8. No complications occurred, and each patient was able to resume their preinjury daily activity and was highly satisfied with their treatment.

**Conclusions:**

In the present study, internal plating for bipolar clavicle injury allowed early mobilization and resulted in good joint function. We recommend fixation of the more severely affected side first because the other side may be passively reduced and acquire stability once the more severely affected side has been fixed. Internal fixation of the other end may therefore be unnecessary unless residual instability exists.

**Supplementary Information:**

The online version contains supplementary material available at 10.1186/s12891-023-06126-1.

## Background

The clavicle is a double-curved S-shaped bone, and it is the only long horizontal bone connecting the axial and upper girdle bones. Clavicle fractures are very common injuries, representing 2.6% to 4.0% of all fractures, and up to 82% of clavicle fractures occur in the middle third of the clavicle [1, 2]. By contrast, lateral clavicle fractures and medial clavicle fractures account for 28% [3] and 3% [1] of all clavicle fractures, respectively. Sternoclavicular (SC) joint dislocation and acromioclavicular (AC) joint dislocation account for approximately 3% [4] and 9% [5] of shoulder girdle injuries, respectively. However, injury of both ends of the clavicle is extremely rare [6, 7]. This injury is also called “bipolar clavicle injury” or “floating clavicle” [8, 9]. In 1831, Porral [10] reported the first case of dislocations of both the AC and SC joints. Beckman [11] reported the 16th case in 1924. Additional cases were not reported until 1982 [12]. Bipolar clavicle injury is currently defined as any combination of dislocation and/or fracture at both ends of the clavicle [6–8].

Bipolar clavicle injury is usually the result of high-energy trauma, and most patients have associated injuries such as brain trauma, rib fracture, hemothorax, pneumothorax, scapula fracture, or chest injury [7]. Although road traffic and sports injuries have increased the frequency of clavicle injury, most reports on bipolar clavicle injury have been based on single cases; only a few English-language reports have described multiple cases [7, 8, 13, 14].

Because of the rarity of and limited experience with this injury, treatment remains controversial and challenging. In the early stage, most authors treated their patients nonoperatively with satisfactory results [11, 12, 15, 16]. However, Sanders et al. [17] reported that among six patients with bipolar clavicle injury who were initially treated with conservative methods, four required additional surgical intervention because of continuing pain, and good results were finally obtained after AC joint reconstruction. Additionally, Lee et al. [18] reported superior results in patients who underwent surgical treatment. We herein report seven bipolar clavicle injuries with different injury patterns that were successfully treated by open reduction and internal fixation, and we introduce the technique of using a hook plate for management of SC joint injuries. This study was performed to assess the effectiveness and safety of internal plating for bipolar clavicle injuries and to review recently published literature.

## Methods

### Patients

We retrospectively analyzed patients with clavicle injuries treated from May 2013 to June 2021 in Xi’an Honghui Hospital. This study was conducted according to the Declaration of Helsinki and approved by the Ethics Committee of our institute. All patients provided written informed consent to publish their clinical data and accompanying images. In this study, bipolar clavicle injury was defined as any combination of traumatic dislocation and/or fracture at both ends of the clavicle. The inclusion criteria were skeletally mature patients with bipolar clavicle injuries treated by open reduction and internal plate fixation with a follow-up of more than 12 months. The exclusion criteria were patients younger than 18 years, patients with dislocation and/or fracture of only a single clavicle end, patients with bipolar clavicle injuries treated conservatively, and patients with bipolar clavicle injuries who were followed up for less than 12 months after surgery. A time from injury to surgery of less than 3 weeks was defined as acute injury, whereas a time longer than 3 weeks was defined as old injury. In addition to a preoperative plain radiograph containing the whole length of the clavicle, a computed tomography scan and three-dimensional reconstruction was performed for each patient; this was helpful to evaluate the precise displacement of each end of the clavicle and to establish a preoperative plan. Magnetic resonance imaging was not routinely performed unless there was not enough evidence to assess joint dislocation on the computed tomography scan.

**Fig. 1:**
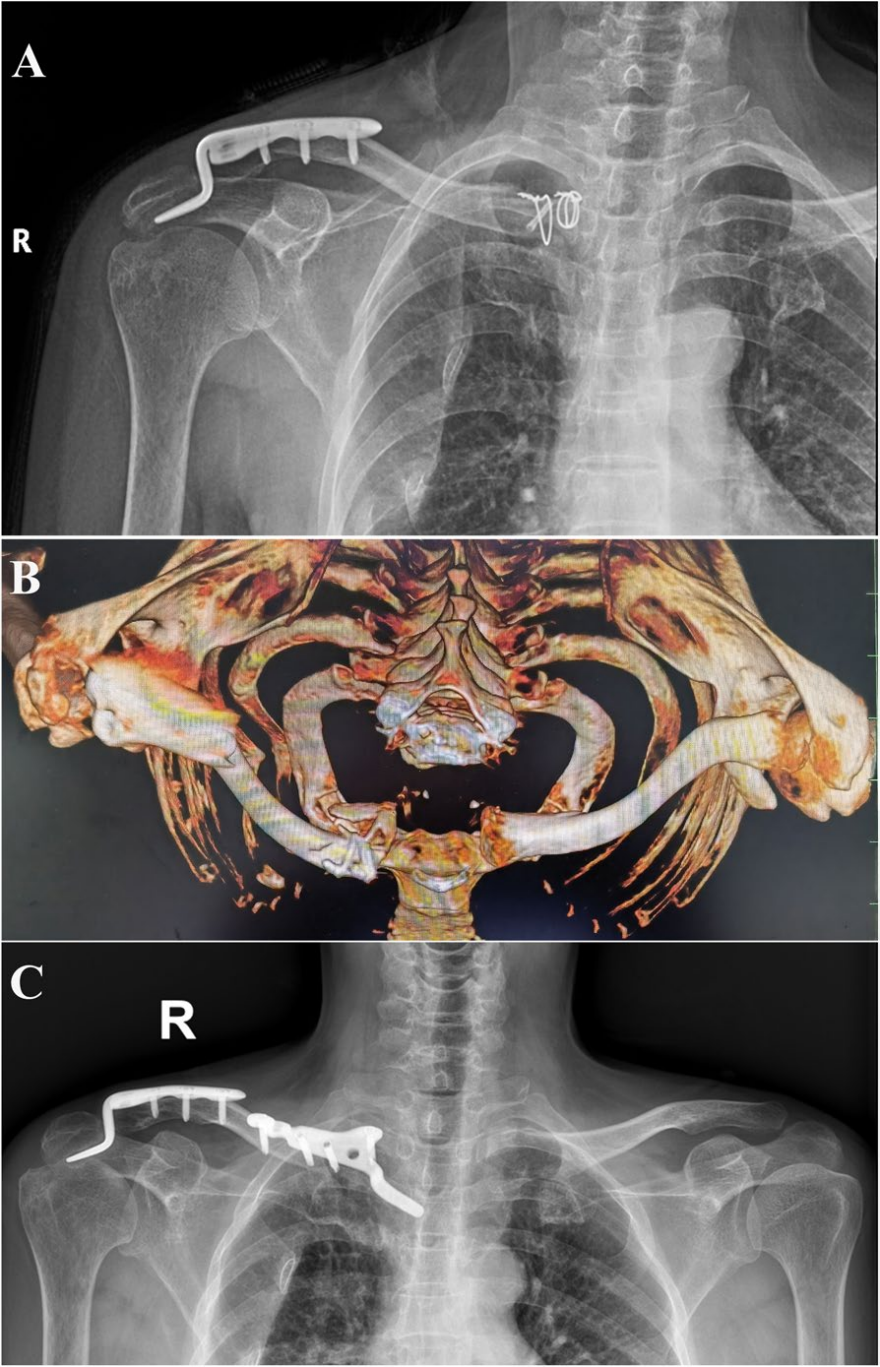
A 62-year-old woman (Patient 5) underwent both medial and distal end fixation in local hospital. She experienced continuous medial clavicle protrusion and pain, and a (**A**) posteroanterior radiograph and (**B**) three-dimensional computed tomography reconstruction from above taken 1 month after surgery showed re-displacement of the medial clavicle bone fragments. She underwent a revision operation involving removal of the initial implant and fixation with a clavicle hook plate. **C** At the 6-month follow-up, the patient showed radiographic congruency of both the sternoclavicular joint and acromioclavicular joint

**Fig. 2:**
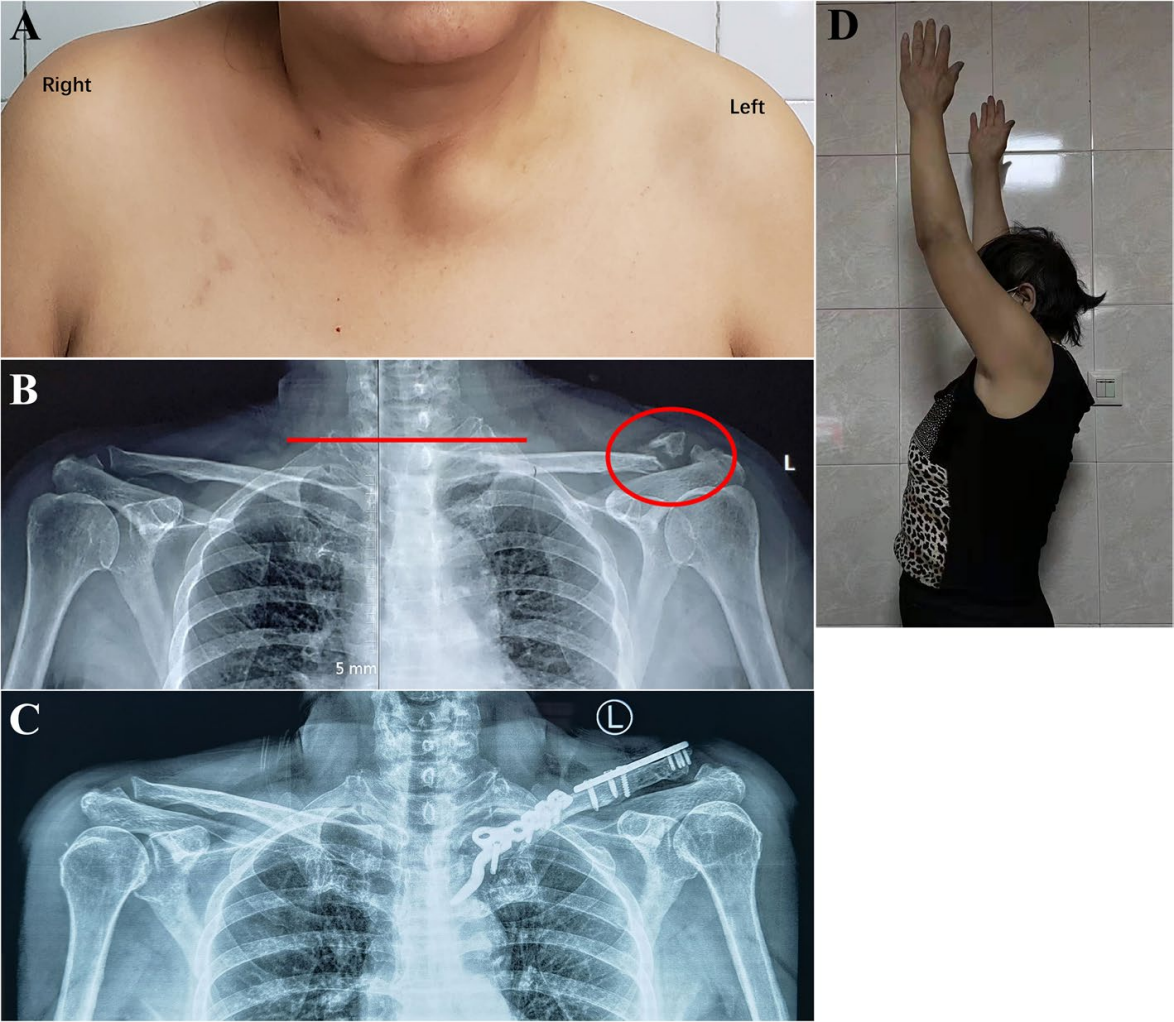
A 58-year-old woman (Patient 3) was injured in a car accident. After repair of the life-threatening injury, she presented to our institute for surgical intervention because of intolerable pain in her shoulder. **A** An anterior view radiograph showed anterior dislocation of the left sternoclavicular joint. **B** A preoperative radiograph showed left anterior dislocation of the sternoclavicular joint (SCJ) and an ipsilateral Neer type II distal clavicle fracture. At the 37-month follow-up, **C** a plain radiograph showed normal alignment of the SCJ, solid union of the distal clavicle, and slight persistent acromioclavicular joint subluxation. **D** Although she had mild shoulder function limitation, she was able to return to her preinjury job

During the study period, we treated a total of 1946 patients with clavicle injuries, among whom 10 patients were diagnosed with bipolar clavicle injuries (all patients’ data and images are provided in the main text (Figs. 1 and 2) and supplemental files (Figure S1-S8)). Two patients who were treated conservatively and one patient who received surgical management with a follow-up period of less than 12 months were excluded from this study. Thus, seven patients with bipolar clavicle injuries treated with internal plate fixation met the inclusion criteria of this study (details are shown in Table 1).

**Table 1:** Patients’ data

Patient	Age (years)	Sex	Side	Mechanism	Associated injury	Medial injury	Lateral injury	Time to surgery(days)	Treatment	Follow up(months)	VAS	Shoulder flexion(°)	DASH score	Constant-Murley	Patient Satisfaction	Complication
1	54	F	R	Fall from height	None	Anterior dislocation	Type IV dislocation	4	SC: hook plate AC: neglected	13	4	150	18.3	72	8	None
2	26	M	L	Crashing	Tooth fracture	Type 1B2 fracture-anterior dislocation	Type IV dislocation	8	SC: hook plate AC: neglected	14	0	165	3.3	96	10	None
3	58	F	L	Car accident	Chest injury, brain injury	Anterior dislocation	Type II fracture	147	SC: hook plate AC: plate	37	1	160	10	90	9	None
4	47	F	L	Motorbike accident	None	Anterior dislocation	Type I fracture-Type III dislocation	5	SC: neglected AC: hook plate	15	0	165	6.7	94	10	None
5	62	F	R	Car accident	Chest injury, ipsilateral scapular fracture	Type 1B2 Fracture	Type II fracture	32	SC: revision with hook plate AC: hook plate	14	2	150	11.7	88	9	None
6	29	M	R	Fall from height	Chest injury, ipsilateral scapular fracture	Type 1B2 fracture- Anterior dislocation	Type III dislocation	12	SC: hook plate AC: hook plate	31	0	155	6.7	90	9	None
7	64	M	L	Fall from height	Ipsilateral scapular fracture	Type 1B1 fracture	Type II fracture	10	SC: plate AC: neglected	73	0	170	5	92	10	None

Medial clavicle fracture was based on the Edinburgh classification. Distal clavicle fracture was based on Neer’s classification. Acromioclavicular joint dislocation was based on Rockwood’s classification. *VAS* visual analog scale, *DASH score* Disability of the Arm, Shoulder, and Hand score, *AC* acromioclavicular joint, *SC* sternoclavicular joint, *F* female, *M* male, *L* left, *R* right, *IS* injured side, *CS* contralateral side

The patients comprised four women and three men with a mean age of 48.6 ± 15.4 years. The injury mechanisms were three falls from a height, one crash, two car accidents, and one motorbike accident. Four patients were injured on the left side and three were injured on the right. All patients had closed injuries without damage to neurovascular or mediastinal structures. Five patients had multiple injuries. All these accompanying injures were managed by specialists before treatment by an orthopedic trauma surgeon.

### Surgical technique

The surgical indications were dislocations or fractures that could not be reduced by conservative treatment and/or appeared prone to recurrence after conservative treatment. After administration of general anesthesia, the patient was placed in the beach chair position on the operating table. Priority was given to the more severely affected side at the beginning of the surgery. For treatment of the medial end of the clavicle, an oblique incision was made over the medial clavicle and sternum. Soft tissue flaps were retracted, and the displacement of the medial clavicle and the torn surrounding ligaments were examined. All fibrous tissue from the medial aspect of the clavicle and fracture fragments were debrided. After achievement of SC joint and/or fracture reduction, a 1.5-mm Kirschner wire was used for temporary fixation. Torn ligaments were repaired with nonabsorbable suture, and this joint was then fixed by a plate or a hook plate. If a hook plate was chosen, a space very close to the posterior dorsal-osteal face of the sternal manubrium was then bluntly created with extreme care using a curved hemostatic forceps. The hook of the plate was inserted into the space posterior to the manubrium, and the other end of the hook plate was fixed on the anterior part of the medial clavicle (Fig. 1). If intraoperative examination by C-arm imaging and passive movement confirmed that the AC joints had been passively reduced with no residual instability after fixation of the AC joint, and these injuries were treated conservatively. Otherwise, additional fixation of the lateral clavicle fracture was required after fixation of the SC joint. Next, an incision was made over the lateral aspect of the clavicle to reveal the lateral end of the clavicle. After debridement of fibrous tissue, the fracture fragments or AC dislocation were reduced and fixed by a locking plate or a hook plate. For the AC joint dislocation in Patient 4, a longitudinal incision was made above the AC joint; after debridement, the reduced AC joint was temporarily fixed by a 1.5-mm Kirschner wire. A clavicle hook plate was then inserted under the acromion and fixed, and passive accurate reduction of the SC joint was confirmed by intraoperative imaging; further fixation of the SC joint was not needed. Finally, the surgical wounds were closed in layers.

### Postoperative management

Following surgery, the affected shoulder was placed in a sling for 4 weeks, and active elbow flexion and extension were started immediately after surgery. Codman’s pendulum exercises were gently performed under a physiotherapist’s supervision, and the patients gradually began to perform passive forward flexion, abduction, and horizontal rotation of the shoulder. Resistance exercises began at 2 months, active strengthening exercises began at 3 months, and the patients were permitted to return to their regular activity at 6 months postoperatively.

The patients were followed up at 1, 2, 3, and 6 months postoperatively and every 6 months thereafter. X-rays contained the whole length of the clavicle, including both the SC and AC joints. Fracture union was defined as evidence of at least three of four healed cortices across the fracture site [19]. Upper limb function was evaluated with the Disability of the Arm, Shoulder, and Hand (DASH) score, which ranges from 0 to 100, and a high score indicates a high level of dysfunction. Shoulder function was evaluated using the Constant–Murley score; a score of 86 to 100 points was considered an excellent result, 71 to 85 was considered good, 56 to 70 was considered fair, and < 56 was considered poor [4, 19]. Pain was assessed using a visual analog scale (VAS) ranging from 0 to 10, with a high score representing a high level of pain [20]. Patient satisfaction was graded on an ordinal scale from 1 to 10, with a high score indicating a high level of satisfaction. Intraoperative and postoperative complications were also recorded.

## Results

The prevalence of bipolar clavicle injury in this study was 10/1946. The study cohort comprised seven patients with four different injury patterns: one patient (Patient 1) presented with dislocations of both the AC and SC joints, two patients (Patient 2 and Patient 6) presented with fracture-dislocation of the SC joint associated with AC dislocation, two patients (Patient 3 and Patient 4) presented with SC dislocation associated with a distal clavicle fracture or fracture-dislocation, and two patients (Patient 5 and Patient 7) presented with both medial and lateral clavicle end fractures. In terms of injury classification, each medial end of the clavicle in patients with SC dislocation was displaced anteriorly. Patients 1 and 2 had type IV AC joint dislocation and Patients 4 and 6 had type III AC joint dislocation according to the Rockwood classification [5]. Patients 2, 5, and 6 had a type 1B2 medial clavicle fracture and Patient 7 had a type 1B1 medial clavicle fracture according to the Edinburgh classification [19]. Patient 4 had a type I distal clavicle fracture and Patients 3, 5, and 7 had a type II distal clavicle fracture according to Neer’s classification [3]. Patients 3 and 5 had old injuries.

Six patients were initially treated in our institute. One patient (Patient 5) was initially treated surgically in a local hospital for fractures of both clavicle ends, and revision of the medial clavicle end fracture by a hook plate was applied in our institute 1 month after the injury because of re-displacement of the bone fragments (Fig. 1). Four patients underwent fixation of only one end, and three patients underwent fixation of both ends. Except for slight AC joint dislocation in Patient 3, immediate postoperative plain radiography confirmed correct hook plate placement and accurate reduction of each joint in all patients.

The patients were regularly followed up after the operation. Each patient underwent a final evaluation after a mean follow-up of 28.1 ± 22.0 months, and functional parameters were assessed over time. The data from the last follow-up were used for the final statistical analysis. At the last follow-up, each fracture had solid union, and each dislocation showed no sign of recurrent instability. The mean shoulder forward flexion was 159.3° ± 7.9°, and the mean DASH score was 8.8 ± 5.1 points. The mean Constant–Murley score was 88.9 ± 7.9 points, with six cases assessed as excellent and one assessed as good. The mean VAS score was 1.0 ± 1.5 points, and the mean patient satisfaction score was 9.3 ± 0.8 points. Patients with ipsilateral scapular fractures or chest injury (Patients 3, 5, 6, and 7) achieved outcomes similar to those of patients without associated injuries. Patients treated operatively achieved better outcomes than the two patients treated conservatively (see Supplemental Table 1, Supplemental Figure S6, and Supplemental Figure S7). Although Patients 1, 5, and 6 had mild shoulder limitation, they were able to resume their preinjury daily activity and were highly satisfied with their treatments. Patients 2 and 6 underwent implant removal after bone healing, and the remaining five patients had no plans to remove the internal implant because no discomfort had occurred. No patients developed complications such as rupture of important structures, subacromial impingement, rotator cuff damage, retrosternal pain, infection, hardware failure, hook migration, or vital organ injury.

## Discussion

The mechanism of bipolar clavicle injury remains unclear. This injury is frequently a result of high-energy trauma such as a deforming force on the lateral aspect of the shoulder or a driving force squeezing the shoulders together combined with trunk torsion [8]. Two hypotheses have been advocated. One suggests that two dislocations occur simultaneously, and the trauma force on the shoulder is initially transformed into elastic energy affecting the clavicle. When the external force disappears, the clavicle relaxes and returns to its normal shape; however, the energy continues to be conducted on both sides of the clavicle, causing ligament damage to each clavicle end and subsequent dislocation of the AC and SC joints [21]. The other hypothesis is initial dislocation of the SC joint with subsequent dislocation of the AC joint [15].

Four different bipolar clavicle injury patterns have been reported: dislocation of both ends of the clavicle, dislocation of the SC joint with distal clavicle end fracture, dislocation of the AC joint with medial clavicle end fracture, and segmental fracture of the clavicle [18]. For most floating clavicles, the medial end is displaced anteriorly while the lateral end is displaced superiorly or posteriorly (Rockwood type III or IV). Eni-Olotu and Hobbs [16] reported a case of inferior displacement of the lateral end and superior displacement of the medial end. Only a few bipolar clavicle injuries with posterior SC joint dislocation have been described [7, 8, 22, 23]. Posterior SC joint dislocations and medial clavicle fractures are life-threatening injuries because of their potential to cause damage to retrosternal structures. Among the cases in the present study, we treated two extremely rare cases of AC joint dislocation combined with medial clavicle end fracture-dislocation. To the best of our knowledge, only one such case has been reported in the past few years [18]. We also treated a rare case of anterior SC subluxation and a nondisplaced fracture of the lateral end associated with AC dislocation of the same clavicle (Patient 4); to our knowledge, only one author has reported such a case.

With respect to treatment of clavicle fractures, both surgical and conservative treatment can achieve satisfactory outcomes with a high bone union rate [2, 24, 25]. Pediatric clavicular fractures [26] as well as stable or non-displaced dislocation of the AC joint [5] or SC joint [4] can be successfully managed with conservative treatment. Because most patients with bipolar injury sustained deformity, residual pain, or instability after conservative treatment [15, 16], the consensus seems to lean toward surgical treatment in younger and active patients [7, 18], especially patients with ipsilateral limb injuries. Surgery can provide a lower chance of non-union, a shorter time to return to work, and better limb function.

When bipolar clavicle injury is treated operatively, a surgical approach to the AC dislocation and lateral end fracture is well-described and standard. Surgical options vary from internal fixation (hook plate, Kirschner wires, pins) to ligament reconstruction (Weaver–Dunn procedure, coracoclavicular reconstruction) [3, 5]. However, there is no consensus on the standard treatment strategy for SC joint injury. Surgical treatment is challenging because of the proximity between the SC joint and important retrosternal structures (trachea, esophagus, brachiocephalic veins, brachiocephalic artery, and brachial plexus). Many operative procedures have been described, such as pins, Kirschner wires, T-plate fixation, medial clavicle resection, and ligament reconstruction, and each has its own advantages and disadvantages [4, 19, 20]. Our previous study showed that a clavicle hook plate is a very feasible option for displaced medial clavicle fractures and SC joint dislocation and has several advantages, such as minimal risk of damage to retrosternal structures (no need to drill holes in the manubrium as for plating, and the 4- to 5-cm thickness of the mediastinal retrosternal prevascular space is sufficient for safe hook insertion [27]), dynamic fixation without damage to the SC joint cartilage surface, and improved fixation stability for comminuted medial clavicle fractures [4]. However, a clavicle hook plate has several drawbacks when used for clavicle injury. First, it might not be indicated for posterior SC dislocation because the hook is inserted posterior to the manubrium, which is consistent with the direction of the dislocated proximal clavicle in posterior SC dislocation. Second, hook migration may occur because the plate can effectively prevent anterior–posterior SC dislocation but does not easily prevent sagittal plane dislocation. Third, potential complications include retrosternal pain, pterygoid shoulder, subacromial osteolysis, acromial impingement, and stress fracture [28]. In this study, we fixed five displaced SC joint injuries and three AC injuries with a hook plate, and primary repair of the torn ligaments surrounding the SC joint was performed before fixation of the anterior SC dislocation. The outcomes in this study were very good, and none of the above-mentioned complications occurred. Our treatment outcomes were equal to those reported by Schemitsch et al. [6], who treated two bipolar clavicle injuries with a clavicle hook plate, and both patients attained good outcomes.

The management sequence of bipolar clavicle injury remains controversial. Schemitsch et al. [6] recommended fixation of the more severely affected side first. However, in a study of six operative cases and five conservative cases, Lee et al. [18] fixed AC injuries with a hook plate first and then performed open reduction and anterior SC ligament repair after failed closed reduction of the medial end of the clavicle. Yurdakul et al. [9] and Thyagarajan et al. [22] fixed the SC joint first in their reports. Our experience was consistent with that of Schemitsch et al. [6]: once the more severely affected side was stabilized, the other end was found to be passively reducible.

In this study, one patient (Fig. 2) who preoperatively presented with SC dislocation associated with a distal clavicle fracture sustained slight AC joint dislocation after hook plating in the SC joint and anatomic plate osteosynthesis in the distal clavicle. This might have been caused by inaccurate reduction of the distal clavicle due to an old fracture or hook plating under large stress.

This study has several limitations, including the small sample size, lack of a control group, and short follow-up period. However, considering the rarity of bipolar clavicle injury, these limitations did not influence the results.

## Conclusions

Internal plating was proven to be a safe and effective treatment for bipolar clavicle injury. If the less severely affected side is passively reduced and acquires stability after the more severely affected side is fixed, further fixation may be unnecessary unless residual instability exists. However, this treatment calls for a well-trained surgeon to perform fixation under appropriate stress and avoid damage to retrosternal structures.

## Supplementary Information

**Additional file 1.**

## Data Availability

The datasets used and analyzed during the present study are not publicly available due to ethical reason but are available from the corresponding author upon reasonable request.

## Abbreviations

AC joint: Acromioclavicular joint
SC joint: Sternoclavicular joint
DASH score: Disability of the Arm, Shoulder, and Hand score
VAS: Visual analog scale
