# Supplementary material for: Treatment of bipolar clavicle injury with internal plating: a case series and literature review
Source: BMC Musculoskelet Disord. 2023 Jan 5;24:8. doi: 10.1186/s12891-023-06126-1 (PMC9814211; doi:10.1186/s12891-023-06126-1)
Supplement: Supplementary file 1 — Additional file 1. [file 12891_2023_6126_MOESM1_ESM.doc]

**Supplemental figures and table**

**
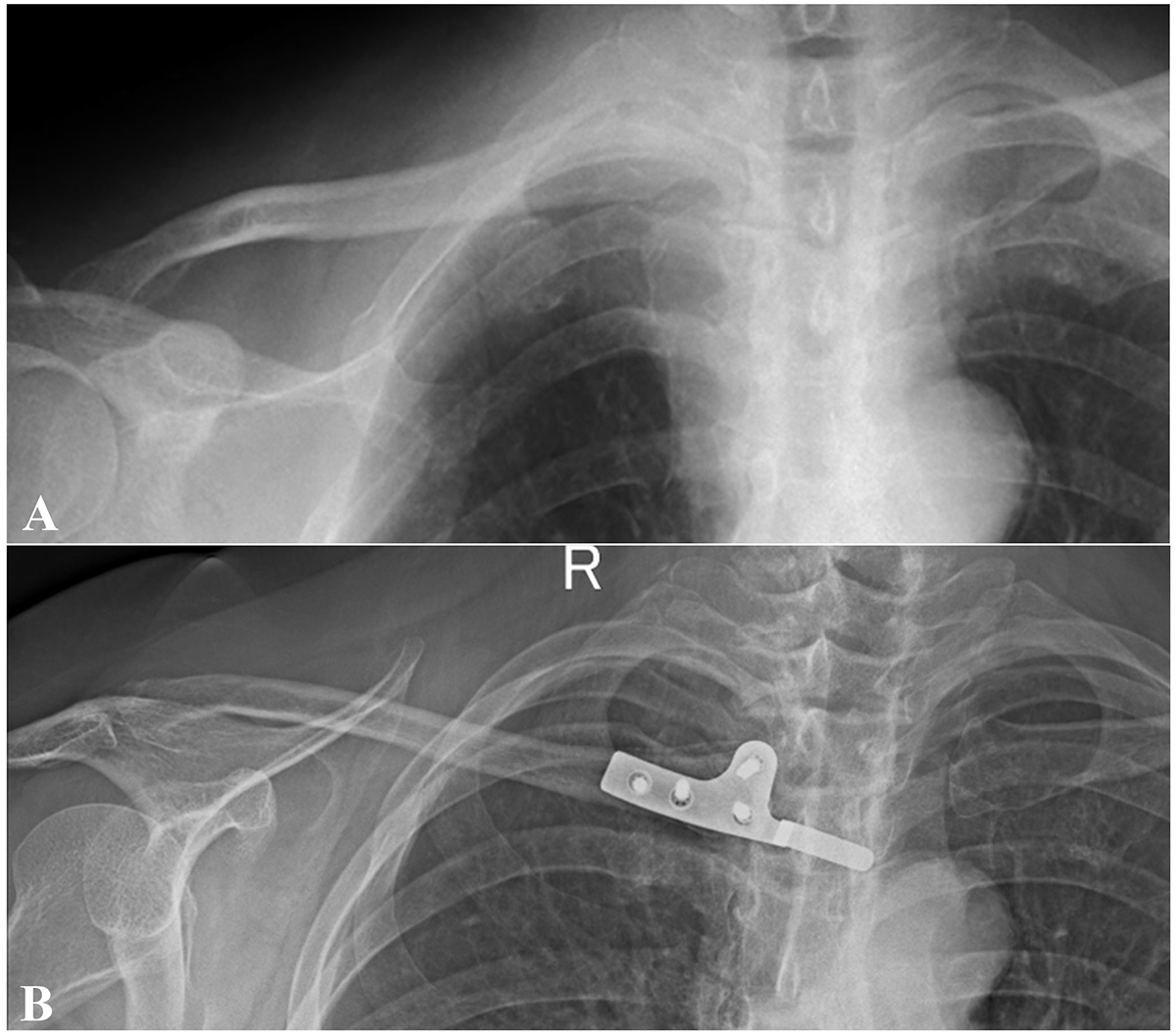
**

Figure S1. Images of a 54-year-old woman (Patient 1). (A) The preoperative radiograph showed right anterior dislocation of the sternoclavicular joint and ipsilateral Rockwood type IV acromioclavicular joint dislocation. (B) A radiograph taken 13 months postoperatively showed good reduction of both the sternoclavicular joint and acromioclavicular joint without implant failure.


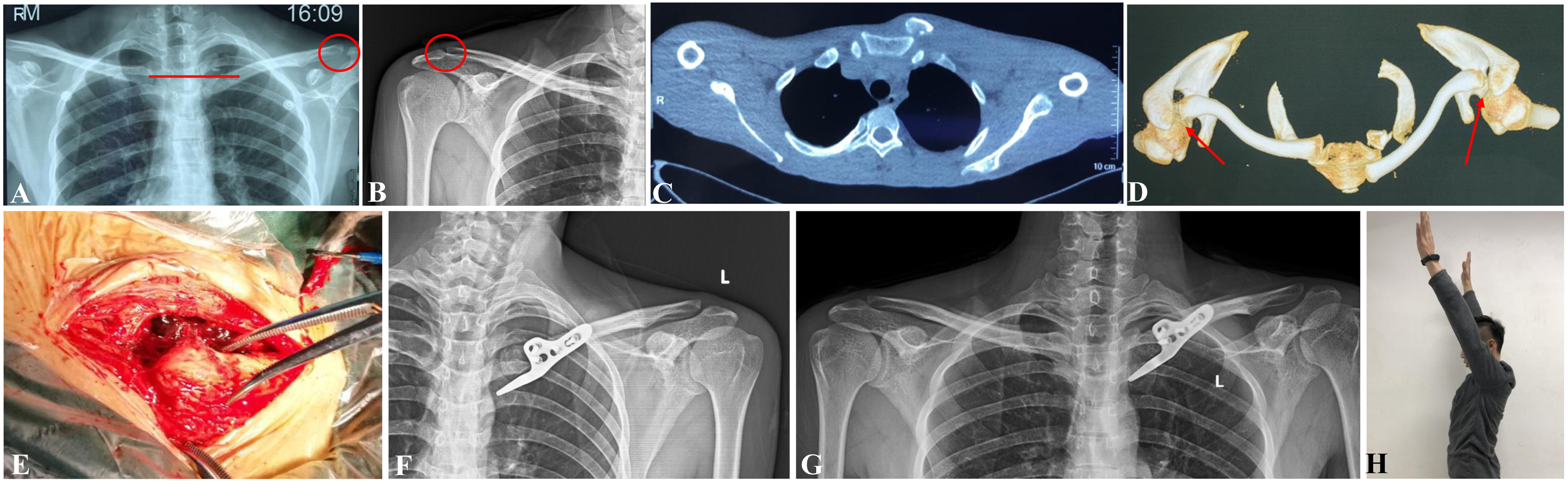


Figure S2. Images of a 26-year-old man (Patient 2). (A, B) The preoperative posteroanterior radiographs showed a dislocated left sternoclavicular joint and slight widening of the left acromioclavicular joint. (C) A computed tomography scan showed intra-articular medial clavicle fracture and impingement of the skin by the medial clavicle. (D) Three-dimensional computed tomography reconstruction from above showed fracture-anterior dislocation of the sternoclavicular joint and sub-dislocation of the acromioclavicular joint. (E) An intraoperative photograph revealed the dislocated medial clavicle end. (F) An immediate postoperative radiograph showed anatomical reduction of both the sternoclavicular joint and acromioclavicular joint as well as satisfactory positioning of the hook plate. At the 8-month follow-up, (G) the patient showed radiographic congruency of both the sternoclavicular joint and acromioclavicular joint and (H) attained full shoulder mobility.


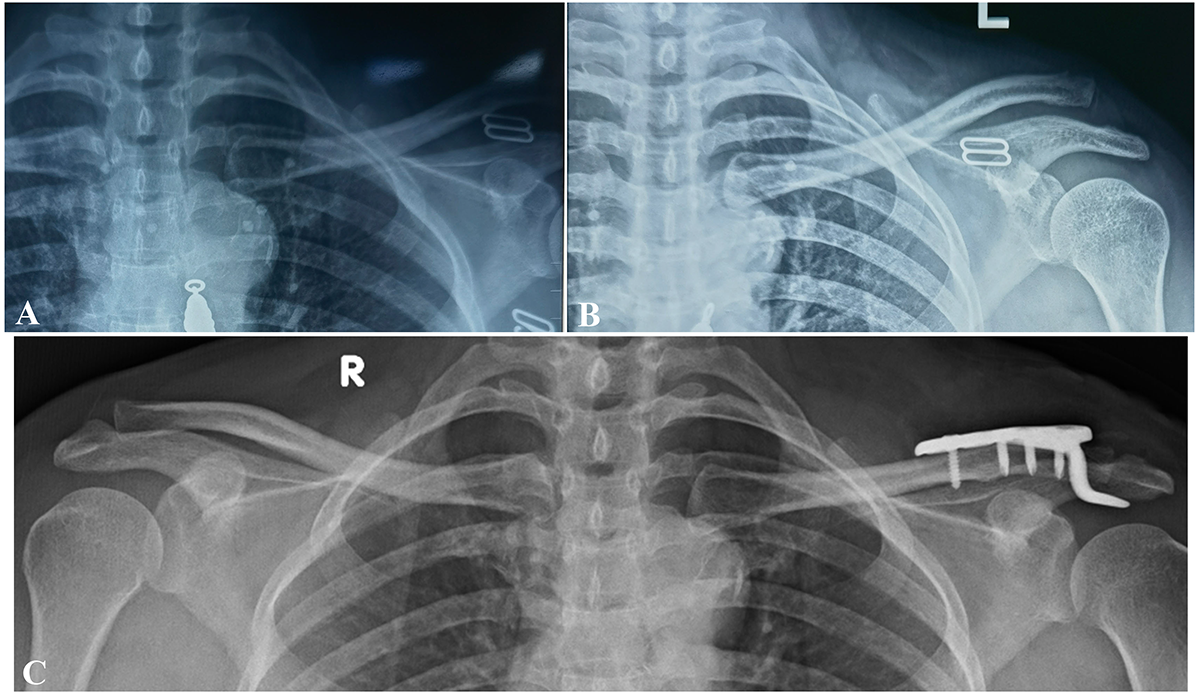


Figure S3. Images of a 47-year-old woman (Patient 4). (A, B) The preoperative posteroanterior radiographs showed slight widening of the left sternoclavicular joint (sub-dislocation) and a dislocated acromioclavicular joint combined with distal clavicle fracture. (C) A radiograph was taken immediately after surgical management of the acromioclavicular joint with a clavicle hook plate.


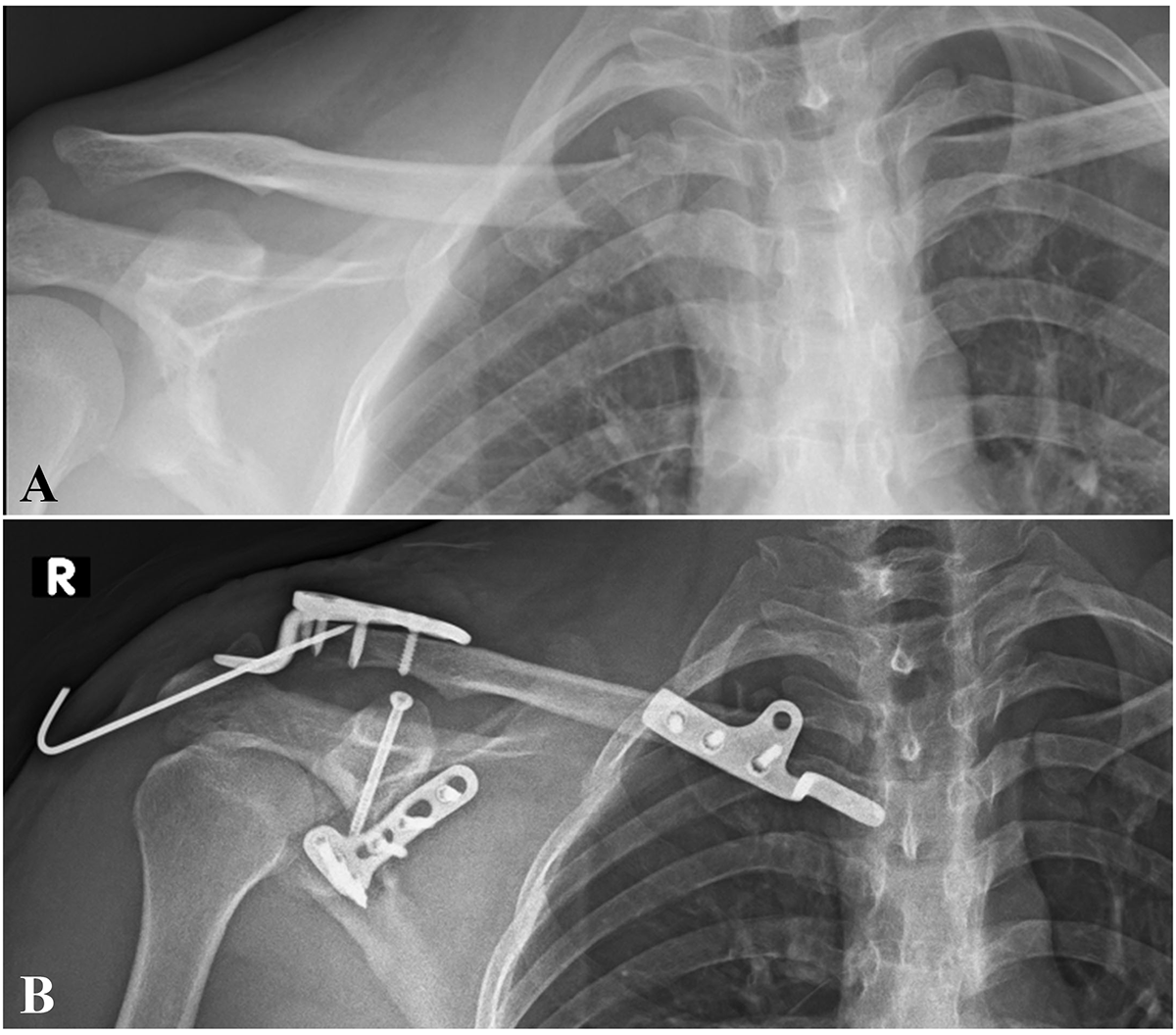


Figure S4. A 29-year-old man (Patient 6) was injured after a fall from a height. (A) He sustained fracture-dislocation of the sternoclavicular joint associated with acromioclavicular joint dislocation, chest injury, and ipsilateral scapular fracture. (B) Both his sternoclavicular joint and acromioclavicular joint were fixed with a clavicle hook plate, and his scapular fracture was fixed with internal plating.


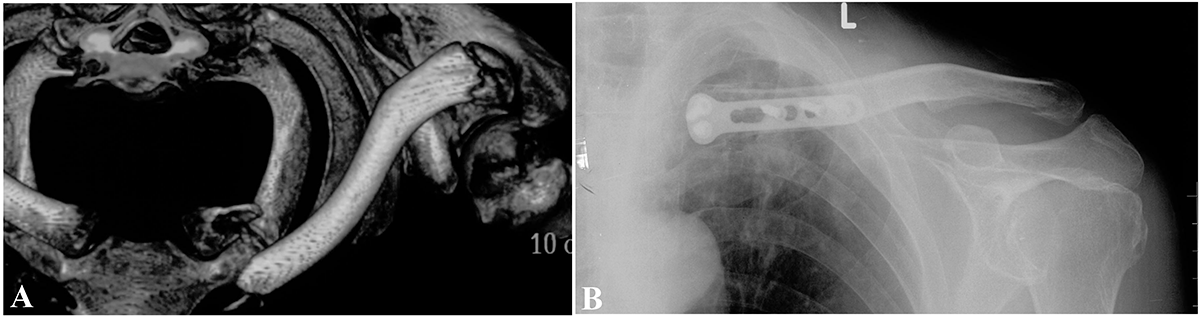


Figure S5. A 64-year-old man (Patient 7) was injured after a fall from a height. (A) A preoperative computed tomography scan showed fractures of both ends of his left clavicle and ipsilateral scapula. (B) A radiograph was taken immediately after surgical management of his left medial clavicle fracture.


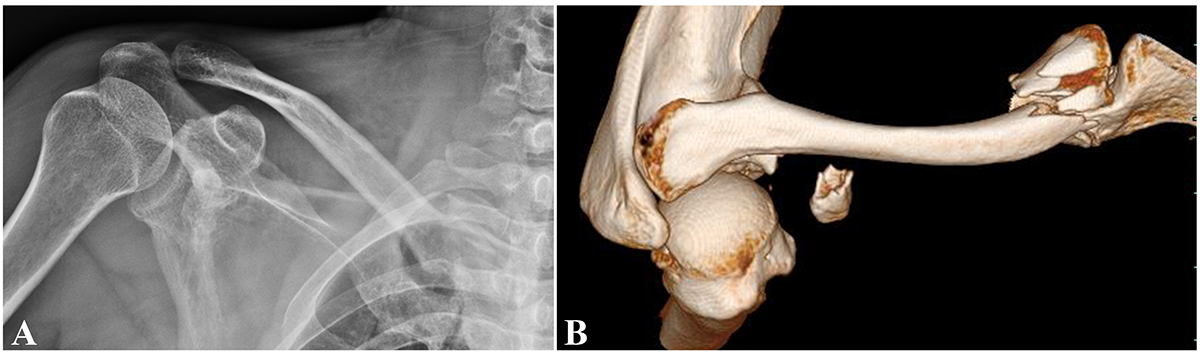


Figure S6. Conservative treatment (Patient 1). A 44-year-old man sustained multiple fractures after a car accident. He refused surgical management of his right clavicle injuries. (A) A radiograph and three-dimensional computed tomography reconstruction showed type Ⅳ dislocation of his right acromioclavicular joint and an intra-articular fracture of the ipsilateral medial clavicle end.


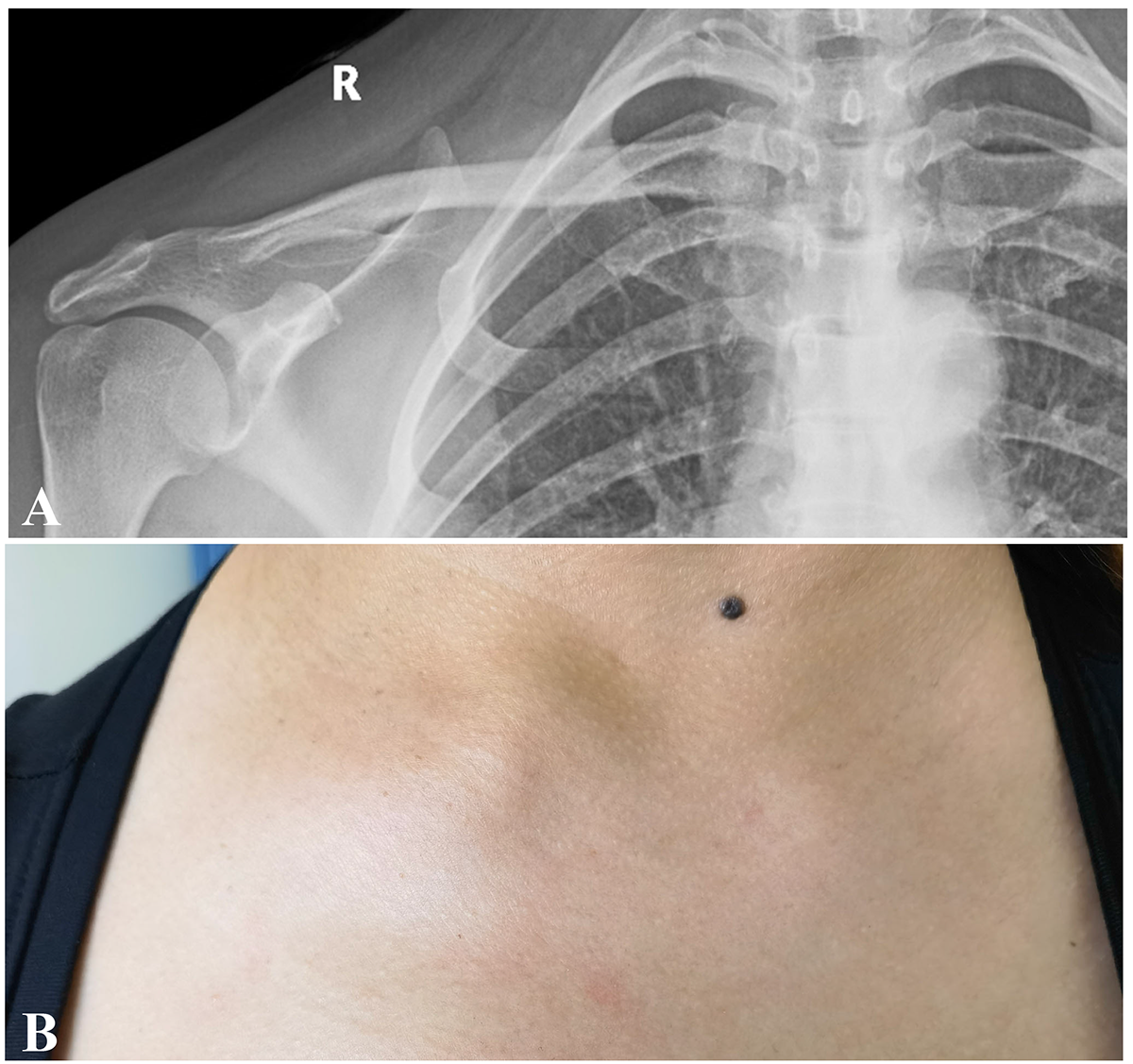


Figure S7. Conservative treatment (Patient 2). (A) A 53-year-old woman sustained type Ⅳ dislocation of her right acromioclavicular associated with anterior dislocation of the ipsilateral sternoclavicular joint. (B) She refused surgical management and had persistent medial clavicle protrusion and pain.


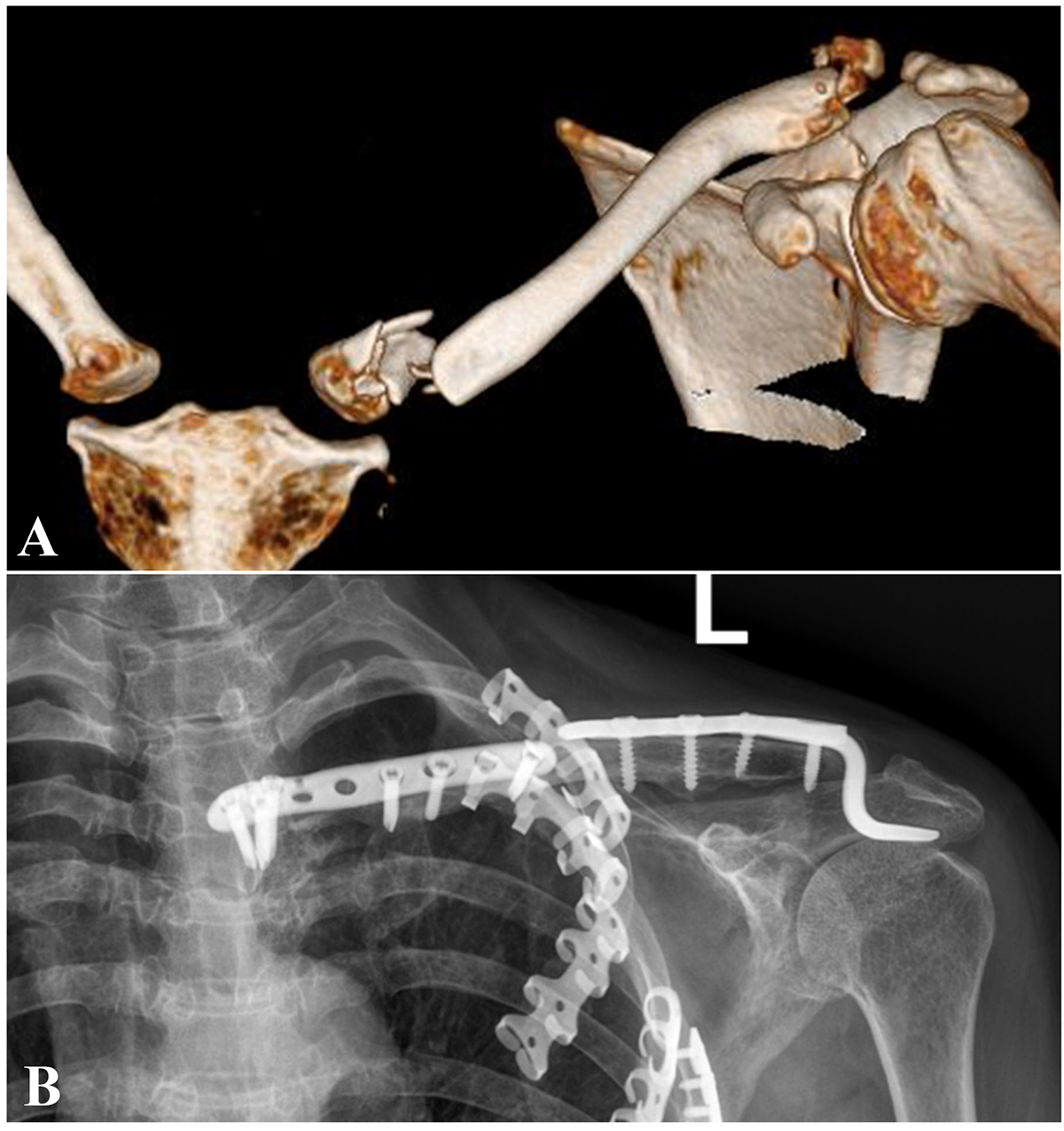


Figure S8. Images of a patient with a follow-up period of less than 12 months after surgical management. A 61-year-old woman sustained a left clavicle fracture, left scapular fracture, chest injury, and pelvic fracture. (A) Three-dimensional computed tomography reconstruction showed displaced fractures of both ends of her left clavicle. (B) A radiograph taken 4 months after surgery showed bone union of both clavicle ends.

Supplemental Table 1. Characteristics and outcomes of patients with bipolar clavicle injury treated conservatively

| Patient | Age (years) | Sex | Side | Mechanism | Associated injury | Medial injury | Lateral injury | Follow up(months) | VAS | Shoulder flexion(°) | DASH score | Constant-Murley | Patient Satisfaction | Complication |
| --- | --- | --- | --- | --- | --- | --- | --- | --- | --- | --- | --- | --- | --- | --- |
| 1 | 44 | M | R | Car accident | Contralateral tibia fractrure, ipsilateral scapular coracoid process fracture, pelvic fracture, lumbar fracture | Type 1B2 fracture | Type Ⅳ dislocation | 25 | 3 | 150 | 15.8 | 75 | 7 | Medial clavicle protrusion |
| 2 | 53 | F | R | Slipping | None | Anterior dislocation | Type Ⅳ dislocation | 15 | 2 | 160 | 5 | 91 | 8 | Lateral clavicle protrusion |

Medial clavicle fracture was based on the Edinburgh classification. Distal clavicle fracture was based on Neer’s classification. Acromioclavicular joint dislocation was based on Rockwood’s classification. VAS, visual analog scale; DASH score, Disability of the Arm, Shoulder, and Hand score; F, female; M, male; R, right.
